# Maladaptive T-Cell Metabolic Fitness in Autoimmune Diseases

**DOI:** 10.3390/cells12212541

**Published:** 2023-10-29

**Authors:** Irene Rose Antony, Brandon Han Siang Wong, Dermot Kelleher, Navin Kumar Verma

**Affiliations:** 1 Lee Kong Chian School of Medicine, Nanyang Technological University, Singapore 308232, Singapore; 2 Vellore Institute of Technology, Vellore 632014, India; irenerose1400@gmail.com (I.R.A.); wong1208@e.ntu.edu.sg (B.H.S.W.); dermot.kelleher@ubc.ca (D.K.); 3 Interdisciplinary Graduate Programme, NTU Institute for Health Technologies (HealthTech-NTU), Nanyang Technological University, Singapore 637335, Singapore; 4 Faculty of Medicine, University of British Columbia, Vancouver, BC V6T 1Z4, Canada; 5 Skin Research Institute of Singapore, Singapore 308205, Singapore

**Keywords:** autoimmunity, glycolysis, LFA-1, metabolites, psoriasis, rheumatoid arthritis, T-cell function, T-cell motility

## Abstract

Immune surveillance and adaptive immune responses, involving continuously circulating and tissue-resident T-lymphocytes, provide host defense against infectious agents and possible malignant transformation while avoiding autoimmune tissue damage. Activation, migration, and deployment of T-cells to affected tissue sites are crucial for mounting an adaptive immune response. An effective adaptive immune defense depends on the ability of T-cells to dynamically reprogram their metabolic requirements in response to environmental cues. Inability of the T-cells to adapt to specific metabolic demands may skew cells to become either hyporesponsive (creating immunocompromised conditions) or hyperactive (causing autoimmune tissue destruction). Here, we review maladaptive T-cell metabolic fitness that can cause autoimmune diseases and discuss how T-cell metabolic programs can potentially be modulated to achieve therapeutic benefits.

## 1. Introduction

T-cells are essential immune cell types and play a key role in adaptive immune responses, implicated in host defense against invading pathogens and antitumor immunity. The two main subtypes—CD4^+^ helper T (Th) cells and CD8^+^ cytotoxic T-cells—continuously recirculate between blood and the secondary lymphoid organs in the naïve quiescent form, requiring minimal metabolic activity. Thus, resting T-cells with low metabolic demands primarily use glucose and fatty acids to generate ATP, utilizing an energy-efficient oxidative metabolism through oxidative phosphorylation (OXPHOS) and β-oxidation.

**Figure 1:**
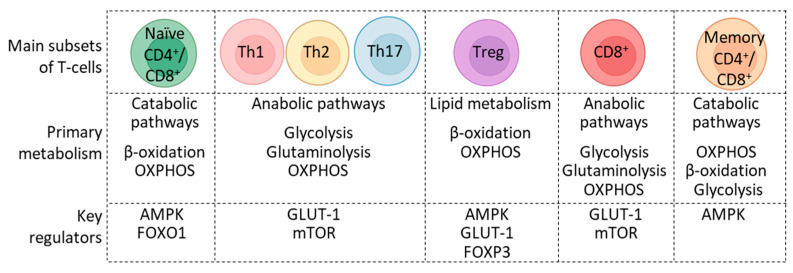
Distinct metabolic programs utilized by specific T-cell subsets in a healthy individual. Key metabolic regulators of various T-cell subsets are listed.

Following antigen recognition and activation, T-cells exit metabolically quiescent state and dynamically remodel themselves into an anabolic and highly energetic effector phase, requiring immunological and microenvironmental signal-dependent rapid fine-tuning of metabolic programs [1]. Helper T-cells differentiate into various effector subtypes, mainly Th1, Th2, and Th17. In order to fuel the increased demands for biosynthesis in effector cells, T-cell activation signals skew cellular metabolic pathways that primarily utilize glucose. These effector cells express high levels of active mammalian target of rapamycin (mTOR), glucose transporter 1 (GLUT-1), glutamine transporters sodium-coupled neutral amino acid transporter 1 (SNAT1) and SNAT2, alanine-serine-cysteine transporter 2 (ASCT2), and enzymes involved in glucose and glutamine metabolism. Increased activities of these proteins and enzymes shift T-cell metabolism from OXPHOS and β-oxidation to anaerobic glycolysis and glutaminolysis. Of note, signaling pathways for enhancing glycolysis vary among effector T subtypes. A subpopulation of helper T-cells, called regulatory T-cells (Tregs), contribute to immune homeostasis by maintaining self-tolerance. Tregs suppress exaggerated immune responses and prevent autoimmunity. Tregs express less GLUT-1 and are less glycolytic than effector T-cells but express high levels of AMP-activated protein kinase (AMPK) and utilize lipid metabolism. Tregs can often be metabolically flexible depending on the microenvironment of the tissue and the availability of nutrients. After antigen clearance, T-cells return to a quiescent state to support T-cell memory function. Although various CD4^+^ and CD4^+^ memory T-cell subsets exhibit varying metabolic profiles, they mainly utilize fatty acid oxidation to produce energy [2,3]. In addition, compared to naïve T-cells, long-lived CD4^+^ memory T-cells have a higher OXPHOS attributed to a higher glycolytic capacity (Figure 1).

Metabolic fitness of T-cells is not only essential to meet their bioenergetic demands for mounting an effective adaptive immune response, but also for determining their fate, antigen-specific activation, clonal expansion, differentiation, cytokine production, motility, and tissue recruitment. These immunological processes are ultimately driven by T-cells adopting an appropriate metabolic state to judicially meet cellular energy requirements. T-cells, at each differentiation state, require appropriate metabolic precursors, metabolism, and energy that are crucial for their effector functions. Dysregulated metabolic adaptation in T-cells or the inability of T-cells to appropriately tune metabolic needs can cause their overactivation, leading to autoimmunity and self-tissue destruction, which is frequently encountered in autoimmune diseases.

Here, we review the maladaptive mechanisms that impact T-cell metabolic fitness implicated in autoimmunity. We discuss abnormal carbohydrate, amino acid, and lipid metabolisms, energy sensors, and age-related metabolic factors that drive T-cell malfunction. Finally, we propose therapeutic strategies to target these abnormalities for managing and treating specific autoimmune conditions.

## 2. Dysregulated T-Cell Carbohydrate Metabolism in Autoimmunity

The most important carbohydrate metabolic adaptation of effector T-cells, immediately after the relay of activation or migration signals, is an accelerated glucose metabolism [3]. For example, following the engagement of the T-cell integrins (such as the lymphocyte function-associated antigen 1 or LFA-1) with the ligands expressed on the inflamed endothelium (e.g., intercellular adhesion molecule 1 or ICAM-1), T-cells are stimulated to migrate [4]. The migration of T-cells is crucial for their entry into lymph nodes and inflamed tissues. The LFA-1/ICAM-1 interactions also tune T-cell functional programs [5,6], which are implicated in the adaptive immune response as well as in autoimmunity. The LFA-1 contact in actively migrating T-cells induces glucose uptake and increases extracellular acidification rates, which result in an increased production of lactate [7,8]. Moreover, T-cell activation signals rapidly increase the expression, activity, and function of proteins and enzymes involved in metabolizing glucose. Specifically, T-cell activation signals increase the expression of glucose transporters (such as GLUT-1), which then translocate from the cytoplasm to the cell surface, increasing cellular glucose uptake and glycolysis.

**Figure 2:**
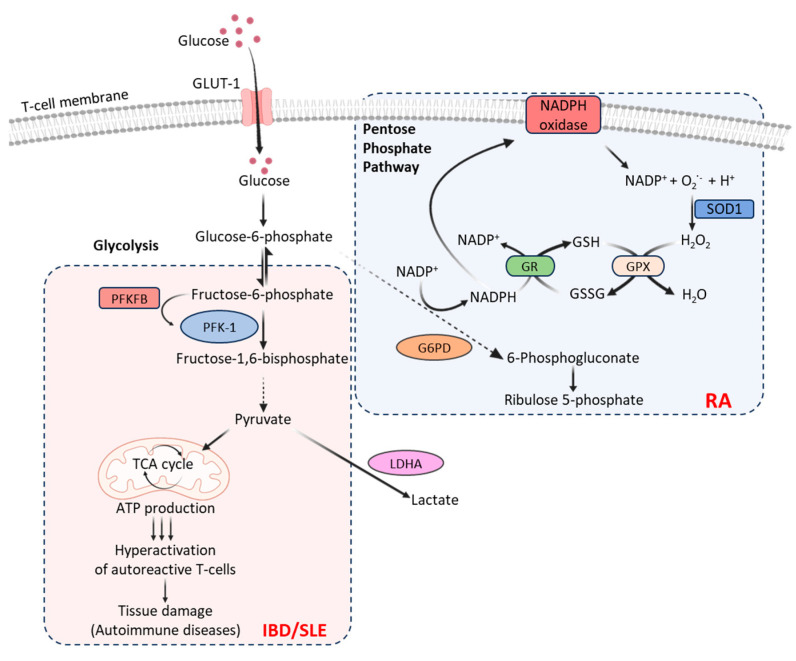
Carbohydrate metabolic pathways in T-cells associated with autoimmune diseases. Following glucose uptake, facilitated by glucose transporters (e.g., GLUT-1), cytoplasmic glucose undergoes glycolysis, producing 2 molecules each of ATP, NADH, and pyruvate from every glucose molecule. Increased glycolysis induces hyperactivation of autoreactive T-cells in patients with IBD and SLE, causing tissue damage. Pyruvate produced by activated T-cells is also converted to lactate with the LDHA enzyme. T-cells in patients with RA metabolize glucose through the pentose phosphate pathway. This pathway generates excessive amounts of NADPH, which converts GSSG into GSH causing diminished intracellular ROS and generation of inflammation-inducing effector cells [Created with BioRender.com, accessed on 1 October 2023].

Autoimmunity-associated T-cells exhibit varying levels of dysregulation for carbohydrate metabolism, depending on the disease’s presentation and T-cells’ bioenergetic demands. For instance, dysfunctional mitochondria in the T-cells of patients with rheumatoid arthritis (RA) produce relatively less lactate and ATP than in healthy individuals [9]. This is because the T-cells of patients with RA metabolize glucose via the pentose phosphate pathway [9] that generates excessive amounts of NADPH, which converts glutathione disulfide (GSSG) into glutathione (GSH) and thus diminishes intracellular reactive oxygen species (ROS) (Figure 2). On the other hand, the T-cells in patients with inflammatory bowel disease (IBD) or systemic lupus erythematosus (SLE) exhibit increased glycolysis (Figure 2) that contributes to proinflammatory responses, causing hyperactivation of autoreactive T-cells and leading to tissue damage [10,11]. The T-cells in patients with hyperplasia-associated diseases, including psoriasis, exhibit uncontrolled cell proliferation associated with increased GLUT-1 levels [12]. These suggest that inhibiting GLUT-1 or T-cell glycolysis with glucose import inhibitors could be exploited as a potential therapeutic approach to limit inflammation and achieve immune tolerance in these autoimmune diseases. Indeed, GLUT-1 inhibition in effector T-cells has been found to reduce disease symptoms in a mouse model of SLE [13].

The rate of glycolysis is controlled by 6-phosphofructo-2-kinase/fructose-2,6-biphosphatase (PFKFB), which is the most potent allosteric activator of the enzyme phosphofructokinase 1 (PFK-1). PFK-1 converts the first glycolysis-committed fructose-6-phosphate into fructose-1,6-bisphosphate and thus regulates cellular glycolytic flux. The PFKFB3 deficiency limits the production of ATP, pyruvate, and lactate, shifting glucose towards the pentose phosphate pathway. Human T-cells express PFKFB3, and its expression is rapidly increased (>20-fold) following T-cell receptor (TCR) engagement and activation signals. The T-cells in patients with SLE and IBD express relatively higher levels of PFKFB3 than that in healthy humans [13]. In patients with RA, T-cells express relatively high levels of glucose-6-phosphate dehydrogenase (G6PD), favoring the pentose phosphate pathway of glucose metabolism [9]. By shifting the PFKFB3/G6PD ratio, the T-cells in patients with RA skew glucose metabolism from ATP production towards NADPH biosynthesis, which generates inflammation-inducing effector cells [9]. In a human tissue–mouse chimera model, the restoration of PFKFB3 expression in adoptively transferred RA T-cells has been shown to reduce synovial tissue inflammation [14].

The monosaccharide carbohydrate *N*-Acetylglucosamine (GlcNAc), a monomeric unit of the polymer chitin and the second most abundant carbohydrate after cellulose, plays an important role in T-cell function [15]. T-cell activation signals trigger the *MGAT-5* gene, leading to *N*-acetylglucosaminyltransferase V-mediated glycosylation of TCR and a consequently skewed T-cell Th1/Th2 balance [16]. Mutated MGAT5 can trigger a hyperimmune response [16], increasing host susceptibility towards IBD with heightened TCR clustering and CD4^+^ T-cell differentiation [17]. The metabolic supplementation of mucosal T-cells from patients with ulcerative colitis (UC) with GlcNAc has been found to enhance branched *N*-glycosylation in the TCR, resulting in the suppression of T-cell growth, inhibition of the Th1/Th17 immune response, and controlled T-cell activity [17]. GlcNAc supplementation therapy alone or combined with anti-inflammatory therapies can therefore be suggested as a potential approach for treating IBD and UC.

Lactate is another important carbon source needed for T-cell metabolic fitness [18]. However, cellular accumulation of lactate in the synovial joints can cause functional impairment of T-cells [19]. The conversion of pyruvate into lactic acid using lactate dehydrogenase A (LDHA) has an important role in safeguarding the proinflammatory and cytolytic effector functions [20]. Thus, remodeling glucose metabolism in T-cells by regulating LDHA activity is an attractive approach to potentially reduce chronic inflammation and autoimmunity.

## 3. Imbalanced T-Cell Lipid Metabolism in Autoimmunity

Lipid species and lipid mediators drive important biological processes in T-cell functional programs, including migration, proliferation, and differentiation [21]. Excessive levels of saturated fatty acids in the extracellular environment skew T-cells towards proinflammatory phenotypes, while polyunsaturated fatty acids promote anti-inflammatory responses [22]. An imbalance in T-cell lipid metabolism and pathways can result in dysfunctional immune processes implicated in autoimmune disorders. For instance, metabolic alterations in the mTORC1 pathway cause intracellular cholesterol accumulation that can hyperactivate CD4^+^ T-cells, leading to autoimmunity [23]. Therefore, inhibiting mTORC1 could be exploited to control hyperactive T-cells as a therapeutic approach in these diseases.

Cholesterol flux is critical in maintaining cellular cholesterol homeostasis. Alterations in the activity of genes involved in T-cell cholesterol uptake, biosynthesis, and signaling (e.g., *LRRD1*, *CYP51A1*, *PASK*, and *Yes1*) have been associated with multiple sclerosis (MS) pathogenesis [24]. Acyl-CoA acyltransferase (ACAT)-1 and ACAT-2 are main cholesterol esterification enzymes that convert free cholesterol to cholesteryl esters, which are stored in the cell [25]. ACAT-1 is expressed in CD8^+^ T-cells and is upregulated upon CD8^+^ T-cell activation [26]. ACAT-1 deficiency due to genetic ablation or its pharmacological inhibition reduces cholesterol esterification but promotes cholesterol biosynthesis in T-cells [26], which can increase cholesterol levels in the T-cell plasma membrane. The cholesterol in the plasma membrane directly binds to the TCR, causing nano-clustering that enhances TCR interactions with a specific antigen, and thus disrupts T-cell homeostasis [27,28]. These altered processes provide a setting for hypercholesterolemia that can lead to the development of T-cell-mediated inflammatory diseases [27,28]. For example, the downregulation of cholesterol biosynthesis enzymes (LDLR, HMGCS1, FDFT1, and DHCR7) has been observed during 25-hydroxycholesterol supplementation (25-HC, an oxysterol known to control cholesterol flux) that aided in suppressing IL-10 in patients with RA [29].

CD4^+^ T-cells can switch from an IFN-γ^+^ effector to an IL-10^+^ anti-inflammatory subtype. Inhibiting the cholesterol biosynthesis pathway with statins during this IFN-γ^+^ to IL-10^+^ switching process has been found to create a block in immune resolution, resulting in a substantial decrease in IL-10 production [30], which is implicated in the pathogenesis of SLE. An altered distribution of sphingolipid/cholesterol-enriched membrane microdomains (lipid rafts) and associated signaling molecules proximal to the antigen receptor have been observed in SLE. These include the reduced expression of the lymphocyte-specific protein kinase (Lck), which regulates T-cell development and homeostasis [31].

In MS, the immune system attacks the lipid-rich myelin sheath of neurons that release a deluge of lipids. A shift from the normal cholesterol biosynthesis pathway to a pathway that produces proinflammatory fatty acids has been observed in patients with MS. The long-chain fatty acids (LCFAs) promote the production of IFN-γ and IL-17 that can exacerbate acute encephalitis, while short-chain fatty acids (SCFAs) exert protection by inducing Forkhead box P3 (FOXP3) expression and thus repressing the differentiation of Th17 subsets implicated in autoimmune reactions [32].

The major autoimmune/autoinflammatory skin diseases, including psoriasis and atopic dermatitis, are characterized by T-cell hyperproliferation in the skin, which depends on cholesterol synthesis and de novo fatty acid synthesis. T-cells with defective fatty acid oxidation will have an impaired function as they are fueled via fatty acid oxidation in the endoplasmic reticulum. The resident memory T-cells present in the skin are functionally specialized in fatty acid uptake from a cutaneous environment. The defect in fatty acid oxidation can be attributed to the promotion of mitochondrial function that increases the proliferation of memory T-cells in T-cell-mediated skin diseases [33,34].

Glycosphingolipids (GSLs) that consist of glycans linked to the C-1 hydroxyl group of ceramides are expressed in cellular plasma membranes. An altered GSL profile influences the balance between stimulatory and inhibitory signals transmitted during T-cell activation, skewing the T-cell function. Peripheral CD4^+^ T-cells in patients with SLE exhibit increased levels of lipid raft-associated GSLs compared to those in healthy individuals. These elevated levels of GSLs in patients with SLE are caused by an increased expression of the nuclear receptor LXRβ, which regulates cellular lipid metabolism and trafficking. LXRβ antagonists can therefore be used to normalize GSL metabolism, correct CD4^+^ T-cell signaling, and restore T-cell function in patients with SLE [35].

## 4. Dysregulated T-Cell Amino Acid Metabolism in Autoimmunity

In addition to glucose and lipid metabolisms, activated T-cells are also dependent on amino acid metabolism. Certain essential amino acids are crucial for the function of T-cells and they serve as metabolic fuels for mitochondrial oxidation and ATP production. For instance, leucine is critical for the efficient activation of T-cells. The L-type amino acid transporter 1 (LAT-1) mediates the transport of amino acids, including tryptophan, arginine, tyrosine, phenylalanine, and leucine. A deficiency in LAT-1 or mTORC1 can result in a dysregulated amino acid metabolism by influencing the fate of T-cell differentiation, thereby causing autoimmunity [36]. Defective amino acid metabolism in T-cells causing hyperactivation of T-cells has been observed in patients with MS [37].

**Figure 3:**
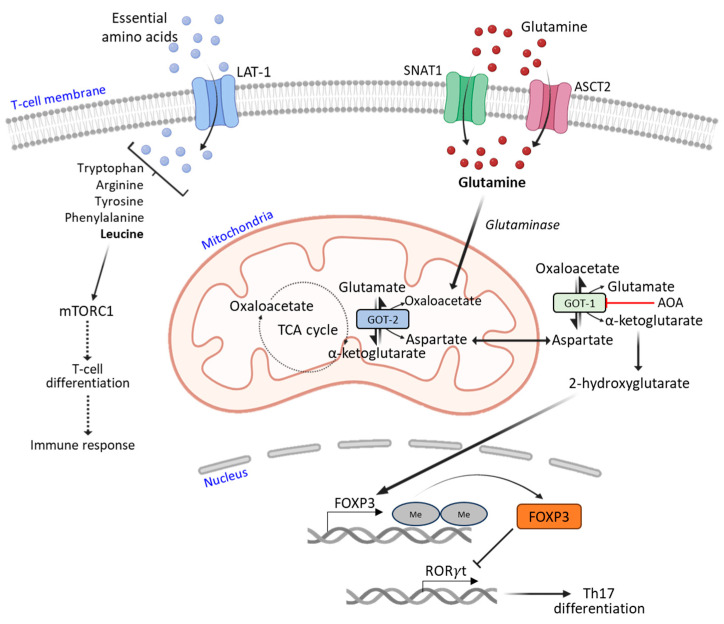
An overview of major amino acid metabolic pathways used by T-cells. Glutamine enters the T-cell cytosol through glutamine transporters, mainly SNAT1 and ASCT2, and subsequently into the mitochondria. It is metabolized into α-ketoglutarate and aspartate, and finally into 2-hydroxyglutarate, which upregulates FOXP3 (which favors Tregs) and downregulates RORγt (inhibiting Th17 differentiation).

T-cells utilize significantly high amounts of glutamine, which is the most abundant amino acid in serum. Once glutamine enters into T-cells through specific amino acid transporters, such as SNAT1 (also known as SLC38A1) and ASCT2 (also known as SLC1A5), it is first hydrolyzed by glutaminase into glutamate. During the process of T-cell differentiation, enzymes glutamate oxaloacetate transaminase 1 (GOT-1) and 2 (GOT-2) present in the cytoplasm and mitochondria, respectively, catalyze the conversion of glutamate and oxaloacetate to α-ketoglutarate and aspartate via a reversible transamination reaction (Figure 3). Since Th17 cells express the only transaminase, GOT-1, these effector cells can therefore be selectively inhibited by the GOT-1-specific inhibitor aminooxy-acetic acid (AOA). Consequently, cellular levels of 2-hydroxyglutarate, a direct metabolic product of α-ketoglutarate, are significantly reduced in differentiated Th17 cells. In addition, 2-hydroxyglutarate reduces the hypermethylation of the *FOXP3* gene, increasing FOXP3 expression, which is one of the major transcription factors that drives and maintains Tregs’ phenotypes and functioning. Increased Tregs exert an antagonistic effect on ROR-γt (a master transcription factor essential for Th17 differentiation), thus repressing Th17 differentiation [38]. Methionine metabolism tunes the T-cell adaptive immune response through the regulation of epigenic reprogramming. In this context, dietary restriction of methionine has been found to reduce the severity of MS disease symptoms by limiting the expansion of Th17 cells in an experimental autoimmune encephalomyelitis (EAE) model [39].

Branched-chain amino transferases (BCAT) are isoenzymes responsible for the first step of the degradation of branched-chain amino acids (BCAAs). The role played by BCAT isoenzymes within the immune cells is still not clear. Among all of the BCAAs, leucine is significantly important, as the loss of BCATc expression can increase the supply of leucine to mTORC1. This can result in T-cell hyperactivation and autoimmunity. A localized depletion of leucine by inducing amino acid-consuming enzymes, such as BCATc, promotes the induction of T-cell anergy [3].

The non-essential amino acid serine, synthesized from a glycolysis intermediate (3-phosphoglycerate), is also important for T-cell proliferation and effector function [40]. In addition, intermediate metabolic products derived from other amino acids, such as nitric oxide (NO) from L-Arginine, mediate changes in T-cell function. For example, NO enhances the differentiation of Th1, Th17, and Th9 cells and therefore exacerbates autoimmune inflammation. Increased NO is a well-established inflammatory factor in RA and IBD. Therefore, limiting soluble NO in the serum could be a potential therapeutic approach to control autoimmunity [41].

## 5. T-Cell Energy Sensors Implicated in Autoimmunity

T-cells utilize energetic and biosynthetic precursors in order to maintain homeostasis and perform effector functions. For example, T-cell motility is regulated with mitochondrial activity and metabolic mediators that precisely co-ordinate receptor–ligand interactions, signal transduction, and cytoskeletal remodeling [42]. An actively migrating T-cell localizes the mitochondria to the uropod to fuel cytoskeletal rearrangements. T-cell energy sensors, such as AMPK, sense changes in energy levels and activate signaling pathways to maintain cellular energy balance. Thus, environmental and immunological stimuli can activate catabolic pathways in T-cells via AMPK to generate ATP. The AMPK pathway cross talks with T-cell lipid and glucose metabolisms [3]. Low energy stores in T-cells activate the AMPK enzyme, which phosphorylates the components of cellular energetic pathways. AMPK also inhibits mTORC1. During low energy levels, AMPK and mTORC1 are connected to each other, and AMPK tends to inactivate mTORC1 to decelerate cellular replication and growth. All of these processes simultaneously restore T-cell’s energy balance for appropriate functions [43]. In patients with RA, T-cells exhibit defective mitochondria and altered recruitment of AMPK, causing hyperproliferation and proinflammatory effector functions [14]. Similarly, T-cells in patients with SLE have dysfunctional mitochondria [44].

*N*-myristylation, a lipid modification process in which myristic acid (a 14-carbon unsaturated fatty acid) is attached to the *N*-terminal glycine of the protein, is required for the lysosomal translocation of AMPK. However, this lipid modification process is defective in RA T-cells due to the reduced concentrations of *N*-myristoyltransferase (NMT1). This causes an increased differentiation rate of T helper cells towards proinflammatory subtypes Th1 and Th17. The loss of the NMT1 enzyme or its genomic alterations causes synovial inflammation and can prevent the lysosomal recruitment of AMPK, resulting in the activation of mTORC1 [45].

In autoimmune skin diseases, T-cells are unable to meet high energy demands, resulting in T-cell dysfunction. While AMPK and mTORC1 are recognized as crucial players in controlling the cellular metabolic state, their detailed mechanisms are not fully understood. The mTORC1 protein can sense the need for metabolic factors (e.g., amino acids), oxygen, energy requirements, growth factors, etc. required for cell division and growth, and can also transmit this information in the regulation of various metabolic processes. However, under hypoxia or ATP limiting inflammatory conditions, mTORC1 is not activated; however, AMPK helps T-cells to adapt to the local metabolic microenvironment [34].

**Figure 4:**
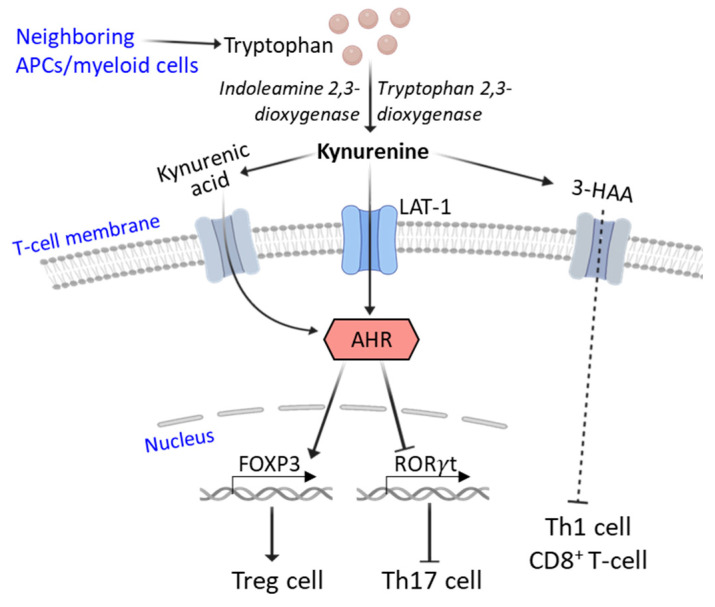
Role of kynurenine and its metabolic intermediates in T-cell function. The kynurenine pathway in APCs and neighboring myeloid cells generates kynurenine and kynurenic acid. These metabolites enter T-cells via the transporter LAT-1, activating the T-cell AHR complex. Activated AHR upregulates the expression of the transcription factor FOXP3, which favors the differentiation and function of Tregs. On the other hand, activated AHR downregulates the expression of the transcription factor RORγt and thus inhibits the differentiation of Th17 cells. An intermediate metabolite of the kynurenine pathway, 3-HAA, inhibits the effector function of Th1 and CD8^+^ T-cells.

The oxidation of tryptophan with antigen presenting cells or neighboring myeloid cells expressing indoleamine 2,3-dioxygenase (IDO) and tryptophan 2,3-dioxygenase (TDO) generates kynurenine, which enters T-cells through the LAT-1 transporter and provides paracrine signaling. Kynurenine and kynurenic acid, which are potent immunomodulatory metabolites, are activating ligands for the aryl hydrocarbon receptor (AHR) complex expressed in T-cells. Kynurenine-induced activation of AHR increases expression of the protein FOXP3, which is a master transcription factor that regulates the differentiation and function of Tregs. On the other hand, kynurenine-induced AHR activation inhibits the expression of retinoic acid receptor-related orphan receptor-γt (ROR-γt), a key transcription factor crucial for the differentiation of Th17 cells (Figure 4). Inhibition of IDO-mediated tryptophan catabolism has been found to exacerbate collagen-induced arthritis in mice and EAE [46,47,48]. Another metabolite of the kynurenine pathway, 3-hydroxyanthranilic acid (3-HAA), has been reported to inhibit Th1/Th17 effector T-cells and promote Tregs, thereby improving EAE, implicated in the regulation of immune responses in patients with MS [49]. In SLE, kynurenine accumulates intracellularly and activates the mTORC1 complex in T-cells skewing the T-cell lineage development. On the contrary, SLE T-cells exhibit diminished cysteine production because of an impaired pentose phosphate pathway. This results in reduced glutathione synthesis and cystine accumulation [50]. Metformin can indirectly inhibit the activation of mTOR and HIF1α and thus reduce Th17 and increase Treg differentiation, decreasing EAE clinical presentations [51].

**Figure 5:**
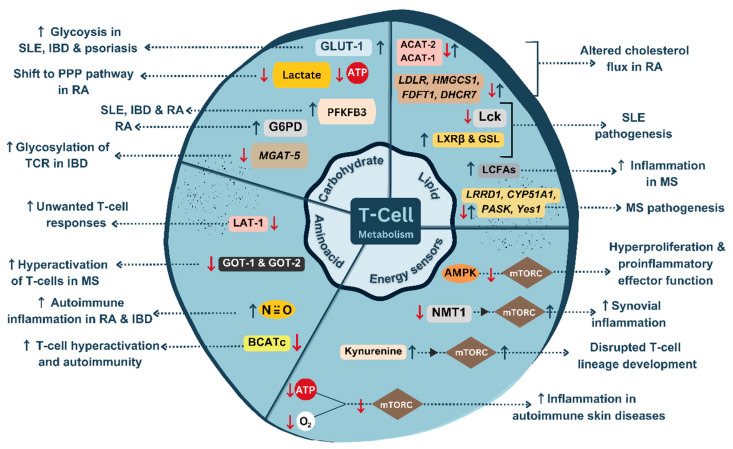
An illustration of dysregulated T-cell metabolism in autoimmunity. ↑, upregulation; ↓, downregulation [created with BioRender.com, accessed on 1 October 2023].

Deficient Tregs, excessive effector T-cells, and the elevated proinflammatory cytokines can induce intestinal inflammation, thereby exacerbating the symptoms of IBD. In IBD, the metabolic sensor AMPK plays an important role in maintaining the gut barrier function and suppressing the inflammation in Tregs. Activation of AMPK can create a pseudo-starving condition that can favor oxidative metabolism and suppress the intestinal inflammation. Additionally, AMPK can alter cellular metabolism by shifting the proinflammatory cytokine production to anti-inflammatory and by promoting T-cell differentiation which supports epithelial barrier function and autophagy. Hence, activating the AMPK can be exploited as a potential therapeutic option for treating IBD. Similarly, another significant metabolic sensor, mTORC1, is highly activated in the gut mucosa of patients with IBD. Thus, inhibiting mTORC1 signaling can also be considered as a potential therapeutic approach for IBD treatment. The mTORC1 inhibitors, including Tacrolimus, Everolimus, and Sirolimus, are being tested in clinical trials for IBD treatment [10]. Mesalamine (5-aminosalicylate), an immunosuppressant which inhibits the mTORC1 signaling, has pleiotropic effects on various energy metabolic pathways, including NF-κB, Wnt/β-catenin, PPAR-γ, MAPK and PI3K/Akt, responsible for colonic inhibition [52]. The metabolic maladaptation occurring in T-cells, which can lead to autoimmunity, are summarized in Figure 5.

## 6. Progressive Deterioration of T-Cell Metabolic Fitness with Age and Autoimmunity

There is a strong connection between ageing and T-cell metabolic pathways. Thymic ageing mainly starts with the T-cells, adapting dynamically to the new organization as the production of new T-cells stops, and the existing T-cells have to deal with the foreign antigens and the chronic infections brought about by it. The other major events in immunological senescence include the damaging of DNA and accumulation of proteins. However, the changes taking place in individuals with specific autoimmune diseases may vary as their T-cells would have already undergone functional transition and become more innate, tissue invasive and proinflammatory in nature [53]. This creates a T-cell aging-associated phenotype (TASP) combined with a gain of new functions and a loss of existing regulatory and cytotoxic effector functions. The TASP can drive tissue inflammation in autoimmune diseases [54].

T-cell homeostatic signaling, mainly the IL-7 signaling, declines with ageing, challenging the metabolic fitness of T-cells. The availability of cytokines and costimulatory molecules can further impact the metabolic profile of ageing T-cells. The young naïve T-cells are constantly exposed to nutrient-rich environments. With ageing, the number of the naïve T-cell population reduces, and the activity of T-cells deteriorates. Trophic signals, including TCR downstream signaling, and homeostatic cytokines regulate the glucose uptake and glycolysis through multiple enzymes. In the absence of these tropic signals, aging-associated T-cells are not able to maintain glucose metabolism, resulting in a reduced mitochondrial membrane potential of T-cells which eventually die. These T-cells give rise to less efficient effector T-cells with an impaired cytokine production [55,56]. However, it remains unclear whether these alterations are the survival adaptations of the aged T-cells, and requires a more detailed investigation to understand the functioning of aged T-cell, which may help to manipulate the metabolism in order to prevent T-cell exhaustion.

The pathogenic T-cells in patients with RA could be a consequence of the premature thymic aging, making it a crucial risk determinant in the case of patients with RA. The shortening of telomeric DNA and the instability of mitochondrial DNA can produce metabolites that can expand T-cell endoplasmic reticulum membranes and induce the production of proinflammatory cytokines. Eventually, these T-cells become tissue invasive and activate the inflammasome. RA T-cells easily transform into mobile T-cells, which can easily enter the synovial tissue and cause tissue inflammation. This rapid transformation of T-cells can be attributed to the cells’ altered lipid metabolism [57].

Mitochondrial malfunction is another feature of aged T-cells. Aged naïve T-cells show a lower calcium influx, impaired calcium buffering capacity, reduction in ATP production, increased ROS generation, and reduced clonal expansion compared to the younger T-cells. These changes can affect T-cell metabolism. Reduced oxidative phosphorylation in aged T-cells could be caused by many factors, for example, the decline in the activity of respiratory enzymes, proton leakage, or a decrease in membrane potential [58,59]. However, mitochondrial malfunction associated with aging and an association with autoimmunity are not well understood. Future studies are necessary to understand the role of mitochondrial metabolism in ageing T-cells, which will pave way to develop therapeutic interventions that can improve the immune responses in aged individuals.

A reduction in the suppressive functions of Tregs can make the host cells susceptible to inflammatory diseases; on the other hand, the loss of the function of Tregs can be the cause of the exposure to an inflammatory environment. Most of the abnormalities that are seen in RA T-cells are connected to the defects in mitochondrial DNA, which can have a direct effect on tissue inflammation. The breakdown of the mitochondrial electron transport chain leads to low levels of metabolites and a reduced ROS production, which creates reductive stress. A decrease in aspartate production, which triggers ER membrane expansion and the production of proinflammatory cytokines, has also been observed [60]. However, how inflammation influences the functions of Treg remains unknown. A better understanding of the thymic ageing mechanism would help to uncover the events that cause tissue inflammation.

## 7. Future Research and Conclusions

The metabolic status of T-cell subtypes varies among patients with various levels of tissue destructive autoimmunity, mainly due to the diverse temporal and spatial coordination of the local metabolite microenvironment. However, several questions pertaining to T-cell metabolic flexibility remain to be answered. How do T-cells dynamically reprogram metabolic states to perform specific effector functions? The questions of whether and how metabolic pathways are compartmentalized to support T-cell function in diverse tissues under varying microenvironments—such as those encountered in primary and secondary lymphoid tissues, and in inflamed peripheral tissues in patients with autoimmune diseases—need further research. A vital function of T-cells is their migration to tissue sites, which is an energetically demanding process. How does a T-cell regulate its metabolic activity to meet specific energy demands for motility? Unravelling the molecular basis of the role of cellular metabolism and T-cell migration will expand our understanding of host immunity and autoimmunity. The roles of several metabolic pathway regulators (such as glycogen synthase kinase 3β (GSK-3β), mTOR, AMPK, and Notch1) and adaptor proteins (such as the centrosome and Golgi localized protein kinase *N*-associated protein (CG-NAP), Talin1, and STAT3) in T-cell motility have been established [61,62,63,64]. Can metabolic modulators be combined with LFA-1-targeted therapeutics for safe clinical outcomes? Since local nutrient availability affects T-cell function, it would be imperative to know how diet and dietary restriction shape T-cell metabolic fitness. Ongoing and future studies should shed light on whether specific aspects of a host’s nutrition can be targeted to tackle autoimmune tissue damage.

Therapeutic approaches to manage and treat autoimmune diseases continue to evolve. At the same time, extensive innovations are being developed to improve clinical outcomes. In this context, targeting T-cell metabolic fitness could be an ideal approach to tackle autoimmunity. There is a need to understand maladaptive mechanisms associated with T-cell metabolic fitness, which are responsible for controlling the T-cell function in a diverse range of autoimmune conditions. Current strategies to regulate T-cell metabolic fitness need to be combined with genetic approaches to selectively manipulate the metabolism of specific T-cell subtypes. Therefore, it is imperative to develop novel techniques and to test them in clinical trials, which aim to control T-cell metabolism, to improve the outcomes of autoimmune diseases.

In summary, this review explains the T-cell metabolic maladaptation involved in the initiation and progression of autoimmune diseases. We draw attention to the identified risk genes that skew T-cell maladaptive metabolic programs. Understanding the myriad ways that can cause metabolic dysfunctions in T-cells can potentially help to devise novel therapeutic strategies as well as to further optimize existing strategies in the treatment of autoimmune diseases.

## Data Availability

Not applicable.
